# Real-Time Video Super-Resolution with Spatio-Temporal Modeling and Redundancy-Aware Inference

**DOI:** 10.3390/s23187880

**Published:** 2023-09-14

**Authors:** Wenhao Wang, Zhenbing Liu, Haoxiang Lu, Rushi Lan, Zhaoyuan Zhang

**Affiliations:** School of Computer Science and Information Security, Guilin University of Electronic Technology, Guilin 541004, China

**Keywords:** video super-resolution, temporal aggregation, deformable convolution, redundancy-aware inference, deep learning

## Abstract

Video super-resolution aims to generate high-resolution frames from low-resolution counterparts. It can be regarded as a specialized application of image super-resolution, serving various purposes, such as video display and surveillance. This paper proposes a novel method for real-time video super-resolution. It effectively exploits spatial information by utilizing the capabilities of an image super-resolution model and leverages the temporal information inherent in videos. Specifically, the method incorporates a pre-trained image super-resolution network as its foundational framework, allowing it to leverage existing expertise for super-resolution. A fast temporal information aggregation module is presented to further aggregate temporal cues across frames. By using deformable convolution to align features of neighboring frames, this module takes advantage of inter-frame dependency. In addition, it employs a hierarchical fast spatial offset feature extraction and a channel attention-based temporal fusion. A redundancy-aware inference algorithm is developed to reduce computational redundancy by reusing intermediate features, achieving real-time inferring speed. Extensive experiments on several benchmarks demonstrate that the proposed method can reconstruct satisfactory results with strong quantitative performance and visual qualities. The real-time inferring ability makes it suitable for real-world deployment.

## 1. Introduction

Video is a widely used multimedia format combining image frames with audio. However, the quality of video often is limited by factors such as capture, storage, and transmission [1]. Video super-resolution (SR) techniques aim to reconstruct high-resolution (HR) frames from low-resolution (LR) counterparts. Similarly, image SR models focus on enhancing the resolution of LR images. Video SR can be seen as an extension of single-image SR, which leverages spatial information along with temporal information from LR frames. It has diverse applications in video displaying [2], video surveillance [3], and satellite imagery [4].

Recently, deep learning-based methods have shown promising performance in video SR tasks [2] and image SR tasks [5]. These video SR models can be categorized into two groups: (1) models without image SR techniques and (2) models incorporating image SR techniques. The first category has to explore alternative approaches for spatial information, such as estimating upsampling filters [6] or task-specific optical flow [7]. Although these methods achieve good performance, they have limited spatial information modeling capacity. In contrast, the second category benefits from image SR insights for spatial reconstruction [8,9,10]. However, they only incorporate specific components from image SR models, which creates a barrier to fully harnessing the potential of well-trained parameters. Thereby, there is room for performance improvement. Different from existing video SR models [11,12] that only incorporate specific components from the image SR model, the proposed method employs a full image SR model for better spatial feature extraction and SR reconstruction. Different from Kappeler et al. [1] and Bao et al. [13], the proposed method pre-trains the image SR model only.

Further, numerous video SR models [8,10,14,15,16] focus on performance improvement. Only a few models [11,17,18] take time consumption into account. Fewer models [19,20] are capable of real-time inference. However, real-time inference is important for online applications, such as displaying. Different from previous work [18] that purges unimportant filters, the proposed redundancy-aware inference algorithm reduces time consumption while maintaining all filters in a video SR model.

In this work, a novel video SR method is proposed to address these limitations. To exploit spatial information, the proposed method incorporates the architecture and well-trained weights of an image SR model as the foundational framework. A fast temporal information aggregation module is introduced to effectively leverage inter-frame dependency. Since moving objects exist in different positions, deformable convolution [21] can effectively extract adjacent frame information. Considering the difference of neighboring frames, the channel attention mechanism [22] can adaptively rescale important features, resulting in effective temporal aggregation. The proposed method achieves real-time inference while providing high-quality SR results. Furthermore, a redundancy-aware inference algorithm is developed to reduce repetitive feature extractions. The experiments on popular benchmarks show that the proposed method delivers solid quantitative performance and visual quality. On the one hand, the use of the pre-trained image SR model reduces the difficulty of training a video super-resolution model. On the other hand, it allows the other module to focus on temporal information aggregation. The redundancy-aware inference algorithm significantly reduces the inference latency, making it suitable for applications that need live video SR reconstruction.

The main contributions of this paper are as follows: (1) A novel video SR model is proposed to fully incorporate a pre-trained image SR model and achieve a trade-off between accuracy and real-time efficiency. (1) A novel video SR model is proposed that can be inferred in real-time while providing high-quality SR video frames. (2) A fast temporal information aggregation module is introduced where deformable convolution is adopted to extract the information of a moving object. The channel attention is also employed for adaptively capturing important information. (3) A redundancy-aware inference is developed for video SR. By avoiding repetitive feature extraction, the computational cost is significantly reduced.

The remainder of this paper is organized as follows: Section 2 discusses related works. Section 3 provides a detailed description of the network architecture and the redundancy-aware inference. Section 4 presents datasets, implementation details, experimental results, and analysis. Finally, Section 5 concludes this paper.

## 2. Related Works

### 2.1. Image Super-Resolution

The image SR problem is a typical ill-posed problem. In 2014, Dong et al. [23] were the first to introduce deep learning into this field. Since then, image SR methods have experienced noteworthy advancements [5]. In 2017, Lim et al. [24] proposed the representative EDSR, which made use of residual learning, eliminated unnecessary batch normalization, and expanded the number of parameters while ensuring stable training. To adaptively rescale features, Zhang et al. [22] developed the channel attention mechanism, which has been successfully employed in RCAN. In 2019, Hui et al. [25] presented IMDN, a lightweight model with a small memory footprint that yielded competitive accuracy and enabled quick inference. More recently, the Transformer, originally introduced in natural language processing [26], has been introduced into computer vision [27]. Consequently, the enhanced Swin Transformer [28] has been adopted in SwinIR [29]. By combining convolutional layers and Swin Transformer modules, the proposed approach captures both local and global dependencies simultaneously, resulting in state-of-the-art performance.

In this study, the IMDN [25] is employed as the foundational framework for the following reasons. A real-time video system must deliver a minimum of 24 frames per second, which is important for ensuring a seamless user experience. IMDN [25] has proven its capability in effectively leveraging spatial information for SR reconstruction with a lightweight design.

### 2.2. Video Super-Resolution

Recently, there has been a growing interest in the video (SR) problem, leading to the proposal of numerous deep learning-based models [2]. Given the need to leverage both spatial and temporal information, effectively handling the input low-resolution (LR) frames becomes crucial. We categorize existing methods into the following groups.

The first category includes methods that utilize optical flow. These methods make use of optical flow to align neighboring frames or features. For instance, VESPCN [11] aligns neighboring frames in a coarse-to-fine manner, while TOF [7] learns a task-specific optical flow. Additionally, DRVSR [14] introduces a carefully designed SPMC layer to register pixels in high-resolution, and Wang et al. [30] directly estimated HR optical flow from LR frames. BasicVSR [12] propagates neighbor features via the optical flow. Although these methods have demonstrated promising results, they suffer from high computational complexity. Moreover, inaccurate optical flow estimation can negatively impact the quality of SR results.

The second category contains methods based on 3D convolutions. Three-dimensional convolution is capable of extracting spatial and temporal information simultaneously from multiple input frames. For example, Kim et al. [31] applied 3D convolutions to capture spatio-temporal dependencies in an end-to-end manner, while DUF [6] incorporates 3D convolutions in densely connected blocks. Isobe et al. [32] fused information from neighboring frames using 3D convolutions, and Li et al. [17] proposed fast spatio-temporal residual blocks for reduced latency. The introduction of 3D convolutions alleviates the reliance on inaccurate optical flows and enables end-to-end training. However, the choice of the kernel size in 3D convolutions requires a trade-off between performance under large motion and computational cost.

The third category consists of methods employing deformable convolutions, which have gained popularity recently. Deformable convolutions were proposed in [21]. The learnable offset enables video SR models to capture objects with motion. For instance, Tian et al. [33] employed deformable convolutions to align neighboring frames, while D3Dnet [34] extends deformable convolutions from 2D to 3D for motion adaptivity and spatio-temporal information modeling. EDVR [8] introduces the Pyramid, Cascading, and Deformable convolutions module for neighboring feature alignment. Unlike optical flow-based methods, deformable convolution-based algorithms do not require optical flow estimation, thereby reducing computational costs and enabling end-to-end training.

In addition, there are attention-based approaches. These methods extract spatio-temporal information via various attention mechanisms. For example, Yi et al. [15] and Li et al. [16] adopted non-local attention. Xiao et al. [35] exploited the temporal difference attention. Wang et al. [36] and Xiao et al. [37] made use of deformable attention. Further, some studies [10,38] have employed self-attention mechanisms for video restoration. The attention mechanism can weigh different features according to the input. This allows a model to pay more attention to the key information, thereby improving its accuracy.

For better performance on video SR reconstruction, the proposed method incorporates both deformable convolution and channel attention. The proposed fast temporal information aggregation is achieved through two stages: spatial aggregation and subsequent temporal aggregation. In the spatial aggregation stage, the deformable convolution is employed to align neighboring features. In order to effectively aggregate information from neighboring video frames, channel attention is used. Further, both stages significantly contribute to reconstruction performance.

## 3. Method

### 3.1. Overall Architecture

**Figure 1:**
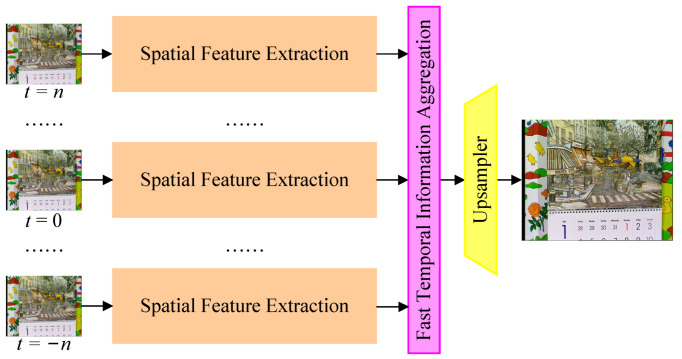
Overall Architecture of the Proposed Method.

The overall architecture of the proposed method is shown in Figure 1. It takes 2n+1 LR frames as input, centered around the target frame to be reconstructed at t=0. The 2n represents the number of neighboring frames. The relative frame index is noted as *t*. The model consists of three key components, i.e., the spatial feature extraction module, the fast temporal information aggregation module, and the upsampler module. The spatial feature extraction module is based on a pre-trained image SR model called IMDN [25]. The fast temporal information aggregation module aligns and fuses neighboring frame features to exploit inter-frame dependencies. Finally, the upsampler module upscales the fused spatio-temporal representation to generate the SR output frame.

**Figure 2:**
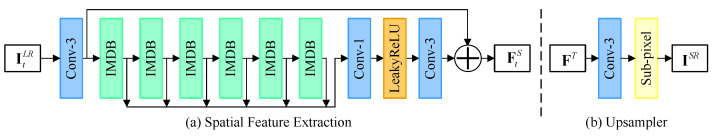
Details of the Spatial Feature Extraction Module and Upsampler Module.

Figure 2a illustrates the spatial feature extraction module, comprising three convolutional layers with varying kernel sizes and six information multi-distillation blocks (IMDB) from IMDN [25]. Conv-3 and Conv-1 refer to the convolutional layers with kernel sizes of three and one, respectively. Additionally, it incorporates global residual learning and hierarchical feature exploitation. It is the foundational framework of the proposed method and is responsible for capturing effective spatial details from input LR frames.

**Figure 3:**
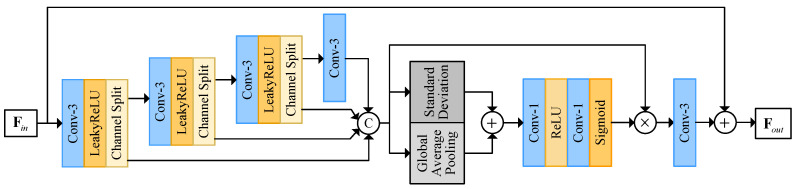
Details of Information Multi-Distillation Blocks Module.

As shown in Figure 3, there are two parts in the IMDB. There are four convolutional layers in the first part. The first three of them are followed by a leaky ReLU and channel split layer. In the channel split layer, the feature is divided into two features. The two features hold 1/4 and 3/4 channels of input feature, respectively. The feature with 1/4 channels is fed to the concatenation. The feature with 3/4 channels is processed by the following convolutional layers. After the concatenation, there is contrast-aware channel attention, which is the second part. It is a more advanced channel attention module that takes not only the average value but also the standard deviation of each feature channel into consideration.

The fast temporal information aggregation module is a key component that allows the model to leverage the inter-frame dependencies. It consists of two stages, i.e., spatial aggregation and temporal aggregation. The spatial aggregation stage gathers information about the same object and aligns it to the center frame. The subsequent temporal aggregation stage fuses information temporally. The details of this module are described in Section 3.2.

Figure 2b shows the upsampler module; it is the final component that converts the fused spatio-temporal features into SR output frames. It contains a convolutional layer and a sub-pixel layer. The convolutional layer adjusts the number of channels. Then, the sub-pixel layer upscales these features to target spatial resolution by rearranging elements from the channel dimension into the spatial dimension.

The specific design allows the spatial feature extraction module to extract information in a manner consistent with an image SR model. Consequently, the parameters of the spatial feature extraction module and the upsampler module can be initialized with well-trained parameters from an image SR model. Leveraging the spatial information extraction abilities learned by the image SR model, the utilization of these well-trained parameters enables the proposed model to make more effective use of spatial information from LR frames. Further, the spatial feature extraction module and upsampler module can be easily replaced by any other image SR models.

Given 2n+1 LR frames $I_{t}^{LR}$, the corresponding target HR frame at t=0 is denoted as $I^{HR}$. The super-resolved frame at t=0, $I^{SR}$, can be produced by

(1) $$I^{SR}=Net\left(I_{t=-n}^{LR},\ldots ,I_{t=0}^{LR},\ldots ,I_{t=n}^{LR}\right),$$

where Net(·) represents the proposed model. As illustrated in Figure 1, there are three modules in the proposed model, i.e., the spatial feature extraction module, the fast temporal information aggregation module, and the upsampler module. The proposed model can be further given by:

(2) $$F_{t}^{S}=FE_{spatial}\left(I_{t}^{LR}\right),$$

(3) $$F^{T}=FE_{aggregation}\left(F_{t=-n}^{S},\ldots ,F_{t=0}^{S},\ldots ,F_{t=n}^{S}\right)$$

(4) $$I^{SR}=U\left(F^{T}\right)$$

where $FE_{spatial}\left(\cdot \right)$, $FE_{aggregation}\left(\cdot \right)$, and U(·) denote the spatial feature extraction module, fast temporal information aggregation module, and upsampler module, respectively. To optimize memory usage, the parameters of $FE_{spatial}\left(\cdot \right)$ are shared across inputs with different timestamps. The spatial feature and temporal aggregated feature are represented as $F_{t}^{S}$ and $F^{T}$, respectively. Following previous work [33], the mean square error (MSE) is applied as the loss function for parameter optimization. For a sample from the training set, the loss function of the proposed model is defined as:

(5) $$L\left(\Theta \right)=\frac{1}{N}\sum_{i=1}^{N}{∥Net\left(I_{t=-n}^{LR,i},\ldots ,I_{t=0}^{LR,i},\ldots ,I_{t=n}^{LR,i}\right)-I^{HR,i}∥}_{2}$$

where Θ denotes the learnable parameters of the proposed model. Further, the L2 norm is ${∥\cdot ∥}_{2}$. The index of the sample in a mini batch is represented by *i*.

### 3.2. Fast Temporal Information Aggregation Module

**Figure 4:**
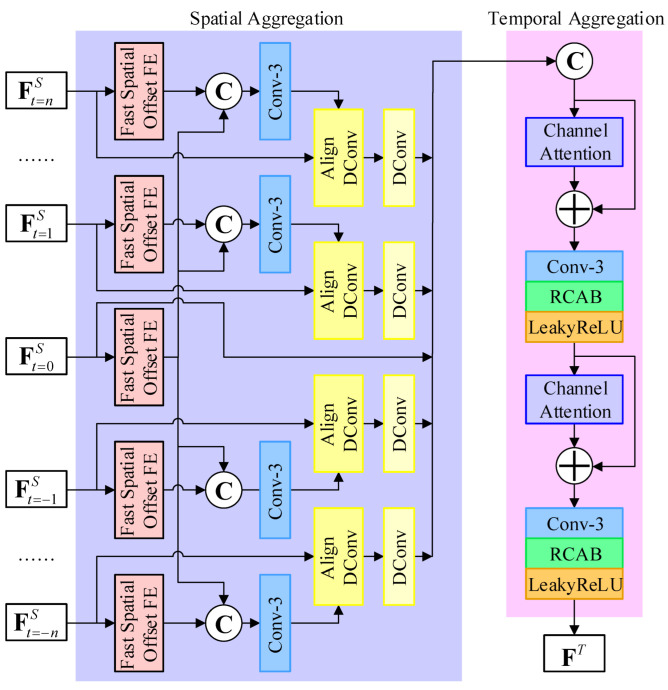
Architecture of the Proposed Fast Temporal Information Aggregation Module.

Figure 4 illustrates the architecture of the proposed fast temporal information aggregation module. The fast temporal information aggregation module aligns and fuses spatial features from the 2n+1 input frames to generate an enriched spatio-temporal feature. It has two stages, i.e., the spatial aggregation stage and the temporal aggregation stage. Thus, the fast temporal information aggregation module can be formulated as:

(6) $$F_{t}^{A}={Aggregate}_{spatial}\left(F_{t}^{S}\right),$$

(7) $$F^{T}={Aggregate}_{temporal}\left(F_{t=-n}^{A},\ldots ,F_{t=n}^{A}\right),$$

where ${Aggregate}_{spatial}\left(\cdot \right)$ and ${Aggregate}_{temporal}\left(\cdot \right)$ denote the spatial and temporal aggregation stage, respectively. The intermediate spatial aggregated feature is denoted as $F_{t}^{A}$, and $F^{T}$ represents the output of this module.

The spatial aggregation stage includes the fast spatial offset feature extraction (FSOFE), the spatial feature alignment, and the spatial feature refinement. The FSOFE is conducted on the spatial feature $F_{t}^{S}$ to obtain the spatial offset feature $F_{t}^{SO}$. Then, for the spatial feature alignment, the offset feature $F_{t}^{O}$ is estimated using a 3×3 convolutional layer. Following this, a deformable convolution is employed for alignment. Unlike the conventional deformable convolution, this variant incorporates additional features for offset estimation, utilizing $F_{t}^{S}$ for feature extraction and $F_{t}^{O}$ for offset information. Finally, another deformable convolution is applied to refine the results in the aligned feature $F_{t}^{A}$. Note that the spatial feature alignment and refinement are skipped for the center spatial feature. The spatial aggregation stage can be expressed as:

(8) $$F_{t}^{SO}=FSOFE\left(F_{t}^{S}\right),$$

(9) $$F_{t\neq 0}^{O}={Conv}_{3\times 3}\left(Concat\left(F_{t\neq 0}^{SO},F_{t=0}^{SO}\right)\right),$$

(10) $$F_{t\neq 0}^{A}=DConv\left(AlignDConv\left(F_{t\neq 0}^{S},F_{t\neq 0}^{O}\right)\right),$$

(11) $$F_{t=0}^{A}=F_{t=0}^{S},$$

where FSOFE(·), Concat(·), ${Conv}_{3\times 3}$, AlignDConv(·), and DConv(·) represent FSOFE, concatenation, convolution with a kernel size of 3, deformable convolution for alignment, and deformable convolution, respectively. The parameters of FSOFE(·), ${Conv}_{3\times 3}$, AlignDConv(·), and DConv(·) are shared to optimize memory consumption. t=0 and t≠0 denote the timestamps of the input center frame and its neighboring frames, respectively.

**Figure 5:**
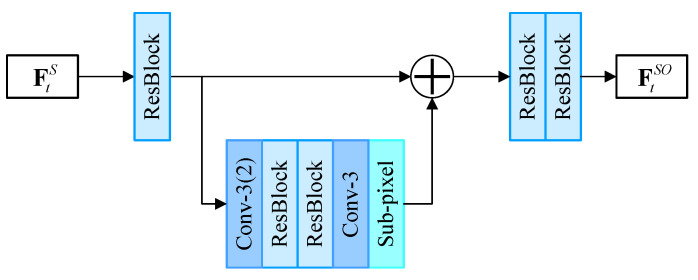
Architecture of Proposed Fast Spatial Offset Feature Extraction.

The FSOFE is responsible for extracting spatial offset features to guide the alignment by deformable convolution. As shown in Figure 5, it adopts a compact two-level hierarchical structure to extract offset efficiently. In the first level, the spatial feature is extracted by a residual block in [24]. In the second level, a 3×3 convolution with stride 2 is applied to reduce the spatial dimensions. The features from these two levels are fused by an element-wise addition and two residual blocks. The output features contain useful offset cues extracted from the spatial features and provide guidance for deformable convolution to adaptively aggregate and align the spatial features from neighboring frames. The two-level design allows the FSOFE to extract offset features with a large receptive field in an efficient manner.

The temporal aggregation stage combines the 2n+1 spatially aligned features $F_{t}^{A}$ to generate a spatio-temporal feature. In order to effectively aggregate useful information, channel attention layer and RCAB [22] are employed. The channel attention adaptively rescales channels within a residual structure. Further, RCAB [22] extracts representative features for reconstruction. Further, a convolutional layer is placed between the channel attention layer and RCAB [22] to reduce the number of channels, resulting in lower inference latency. The optimal architecture of the temporal aggregation stage is provided in Table 1.

Motion among these frames provides valuable cues for reconstructing the center frame. The fast temporal information aggregation module generates a spatio-temporal feature that contains information from all input LR frames. Then, the spatio-temporal feature is upscaled to produce the SR result.

### 3.3. Redundancy-Aware Inference

In order to minimize the computational redundancy that arises during model inference, the redundancy-aware inference (RAI) algorithm is introduced. Considering the fact that once trained, the model parameters remain fixed. As stated in Equation (2), the spatial feature extraction module has to be performed on all neighboring LR frames. However, when inferring consecutive frames in a video, these repeated computations are redundant. This redundancy presents an opportunity to enhance the efficiency and reduce inference latency.

In the standard inference process, which is consistent with the training phase, the spatial feature extraction module is executed 2n+1 times to process each input frame separately. Thus, the latency for inferring a single frame can be expressed as follows:

(12) $$\left(2n+1\right)\times L_{SFE}+L_{FTIA}+L_{U},$$

where $L_{SFE}$, $L_{FTIA}$, and $L_{U}$ are the inference latency of the spatial feature extraction module, fast temporal information aggregation module, and upsampler module, respectively. However, this is redundant as the operations and parameters are identical each time. Hence, some intermediate features, such as $F_{t}^{S}$, remain consistent when generating adjacent SR frames. The RAI reduces this redundancy by caching and reusing these intermediate features. For subsequent frames, the cached features from previous timestamps are reused instead of recomputing them. Only the features from the new input frames need to be extracted. As a result, the latency for inferring a frame after the first *n* and before the last *n* frames can be improved to:

(13) $$L_{SFE}+L_{FTIA}+L_{U},$$

leading to a reduction in latency by $2n\times L_{SFE}$. Similarly, the output of FSOFE, as indicated in Equation (8), can be stored for further processing. Algorithm 1 provides the details of the RAI.

It is important to note that, for simplicity, the processing of the first n and last n frames is omitted. Due to the inconsistency in the processing at both ends, there is a performance degradation in these frames. However, in the proposed RAI, the spatial feature extraction module and the FSOFE are executed once instead of 2n+1 times. It allows for achieving real-time performance during inference without modifying the proposed model.   
**Algorithm** **1:** Redundancy-Aware Inference Algorithm for the Proposed Model.
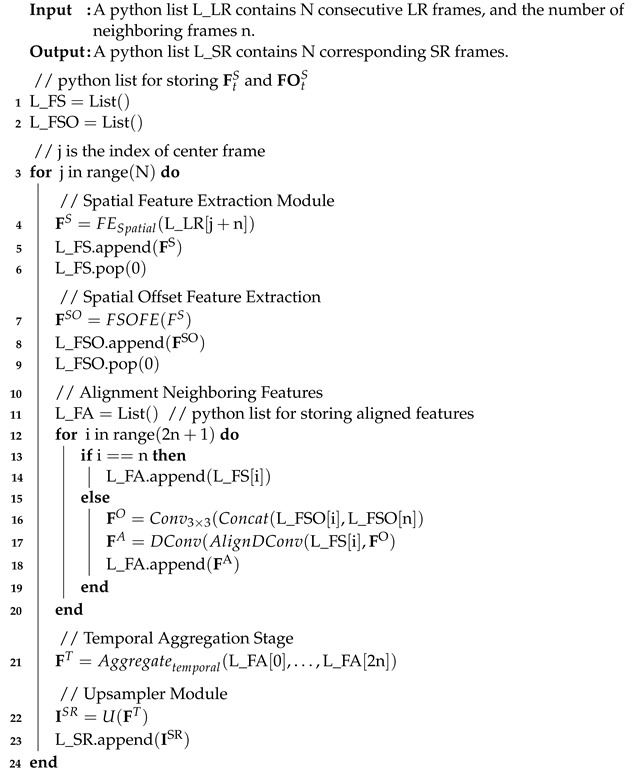


## 4. Experiments

### 4.1. Dataset

In the experiments, Vimeo90K [7] is utilized for training. This dataset contains 64,612 video sequences for training. Each sequence is composed of seven frames. The Vimeo90K dataset has been widely acknowledged and used in various video-related tasks, such as video SR and video interpolation. To evaluate the performance of the proposed model, two well-known benchmarks are employed: Vid4 [33] and SPMCs-30 [14]. The Vid4 benchmark consists of 4 videos with a total of 171 frames. It maintains a minimal resolution of 720×480. In addition to Vid4, the proposed method is evaluated on the SPMCs-30 benchmark, which consists of 30 videos and each video includes 31 frames. The resolution of video frames within the SPMCs-30 is 960×540.

### 4.2. Implement Details

To generate LR frames, bicubic degradation is employed via the Matlab function *imresize*. The downsampling scale factor was set to four. During the training phase, the patch size of the ground truth (GT) and the mini-batch size were empirically set to 256 and 16, respectively. To capture temporal information, the number of neighboring frames is empirically set to two, resulting in the model taking five LR frames as input. Additionally, data augmentation techniques, such as random flipping and rotation, were applied to the training data. The Adam optimizer [39] is utilized to optimize the proposed method, with parameters $\beta_{1}=0.9$ and $\beta_{2}=0.99$. The learning rate was initialized to $1\times 10^{-4}$ and gradually decayed to $1\times 10^{-7}$. The training process lasted for 300,000 iterations. The channel number of the proposed model is empirically set to 64, except for the cases shown in Table 1. All experiments were conducted on a server with Python 3.8, PyTorch 1.12, Intel CPU, and Nvidia 2080Ti GPU.

For initializing the weight of the proposed method, the spatial feature extraction module and upsampler module load the weight of the pre-trained foundational framework, IMDN. The other parameters are initialized by PyTorch. No parameters are frozen when training the proposed method. The training of IMDN is consistent with [25]. The training set for IMDN is DIV2K [40]. The bicubic degradation is adopted to generate LR images. The channel number of IMDN is set to 64. Finally, the batch size for training IMDN is 16.

The performance of the reconstructed frames is assessed by two widely adopted metrics: peak signal-to-noise ratio (PSNR) and structure similarity index (SSIM) [41]. The PSNR of one SR frame is defined as:

(14) $$\mathrm{PSNR}=10\log_{10}\left(\frac{255^{2}}{\mathrm{MSE}}\right),$$

and the mean squared error (MSE) is defined as:

(15) $$\mathrm{MSE}=\frac{1}{P}\sum_{p=1}^{P}{\left(I^{SR}\left(p\right)-I^{HR}\left(p\right)\right)}^{2},$$

where *P* represents the total number of pixels in a frame. $I^{SR}$ and $I^{HR}$ denote the SR frame result and HR frame reference, respectively. Further, SSIM is defined as:

(16) $$\mathrm{SSIM}\left(I^{SR},I^{HR}\right)=\frac{2u_{I^{SR}}u_{I^{HR}}+k_{1}}{u_{I^{SR}}^{2}+u_{I^{HR}}^{2}+k_{1}}\cdot \frac{2\sigma_{I^{SR}I^{HR}}+k_{2}}{\sigma_{I^{SR}}^{2}+\sigma_{I^{HR}}^{2}+k_{2}}$$

where $u_{I^{SR}}$ and $u_{I^{HR}}$ are the mean values of the SR and HR frames, respectively. $\sigma_{I^{SR}}$ and $\sigma_{I^{HR}}$ are the standard deviations of the SR and HR frames, respectively. k1 and k2 are used to stabilize the calculation and set to 0.01 and 0.03, respectively. The covariance of the SR and HR frames is denoted as $\sigma_{I^{SR}I^{HR}}$. Following previous studies [7,19,20,33], these metrics are calculated on the luminance channel (Y channel of YCbCr color space), while cropping the eight pixels near the boundary. Note that all frames were considered for performance evaluation.

### 4.3. Comparisons

For examining the performance of our model, comparisons with one image SR method (IMDN [25]) and six video SR methods (SWRN [19], 3DSRnet [31], TOF [7], EGVSR [20], SOFVSR [30], and RISTN [42]) are conducted. IMDN [25] is a lightweight image SR model and is employed as the foundational framework of the proposed method. SWRN [19] is a novel lightweight video SR method. 3DSRnet [31] is a video SR method that exploits spatial-temporal information via 3D convolution. TOF [7] focuses on estimating task-specific optical flow in videos. EGVSR [20] is a generative adversarial network-based model, and SOFVSR [30] predicts the HR optical flow to enhance video SR results. RISTN [42] leverages temporal features in a recurrent scheme.

**Figure 6:**
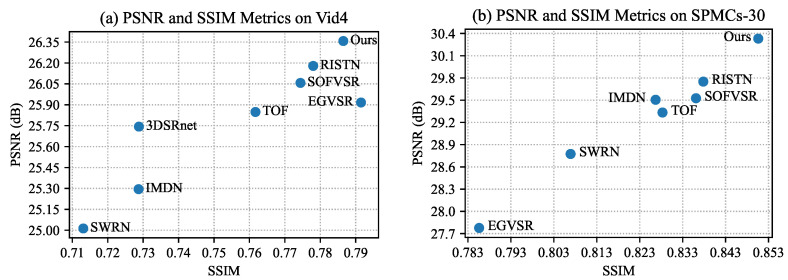
Quantitative Comparison on the Vid4 and SPMCs-30 Benchmarks.

First, the proposed method is evaluated on the Vid4 benchmark. The quantitative results are presented in Table 2 and Figure 6a. In each cell, the first row is the value of PSNR, and the second row is the value of SSIM. The quantitative results on the Vid4 benchmark demonstrate that our method outperforms others in terms of overall performance. Compared with foundational IMDN [25], the proposed method outperforms the PSNR and SSIM metrics by 1.06 and 0.057, respectively. The proposed method is better than the lightweight VSR methods, SWRN [19], and leads by 1.34 dB in PSNR metrics.

In addition, the proposed method is superior to TOF [7] and SOFVSR [30], which are VSR methods based on optical flow. Further, the performance of recurrent-based RISTN [42] is lower than the proposed approach. When compared with GAN-based EGVSR [20], the proposed method underperforms EGVSR on Calendar and City videos but outperforms EGVSR on Foliage and Walk videos. On average, the PSNR value of the proposed method is 0.44 dB higher than EGVSR [20], but the SSIM value is 0.005 lower. Thus, the proposed method demonstrates overall better performance due to its utilization of image SR models, which are excellent at exploiting spatial information. Further, the proposed fast temporal information aggregation module effectively leverages information from neighboring frames. Importantly, the inclusion of the proposed RAI did not negatively impact performance, with only a little degradation of 0.0093 dB and 0.0007 in terms of PSNR and SSIM, respectively.

**Figure 7:**
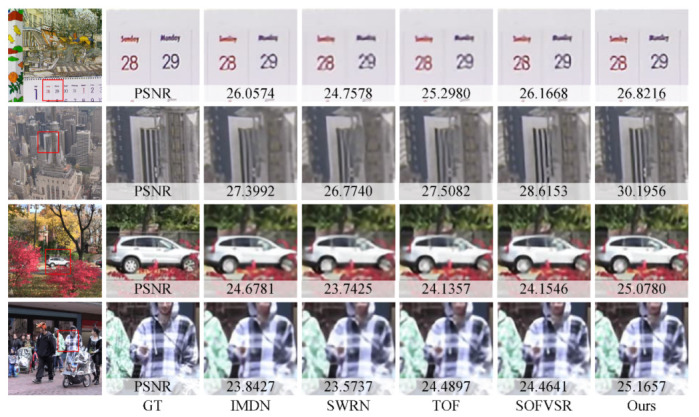
Qualitative Comparisons on the Vid4 Benchmark.

For a qualitative comparison, the proposed method is compared with IMDN [25], SWRN [19], TOF [7], and SOFVSR [30]. As shown in Figure 7, frames from each video are presented, arranged from the top row to bottom as follows: Calendar, City, Foliage, and Walk. In addition, the first column is in the whole frame, the second column labeled GT is a reference to the compared patch, and the third through seventh columns are the results of different methods. The results of each method are marked with the PSNR. Notably, the proposed model delivers superior performance in terms of enhancing text clarity in the Calendar and improving the car’s boundaries in the Foliage. This can be attributed to our model’s utilization of an image SR model as its foundational framework, which gains the capacity to effectively extract and utilize spatial information. Additionally, the proposed method has good performance at reconstructing clear textures of buildings in the City. In Walk, the rope on the clothes is significantly more recognizable. In both of these scenarios, the aggregation of temporal information plays an important role in achieving these improved results.

In addition to the Vid4 benchmark, comparisons on the SPMCs-30 [14] benchmark are conducted. The quantitative results are presented in Table 3 and Figure 6b. On the SPMCs-30 benchmark, the proposed method surpasses all others in terms of average PSNR and SSIM metrics. Specifically, our method exhibits a remarkable improvement of 1.5 dB and 4.3% over SWRN [19] in terms of average PSNR and SSIM, respectively. Compared with optical flow-based methods, TOF [7] and SOFVSR [30], the proposed method outperforms by a margin of 0.8dB in terms of PSNR. Further, the recurrent-based RISTN [42] underperforms compared to the proposed method by 0.58 dB and 0.012 in terms of PSNR and SSIM. Thus, the proposed method makes better use of neighboring information than the recurrent scheme in RISTN [42].

**Figure 8:**
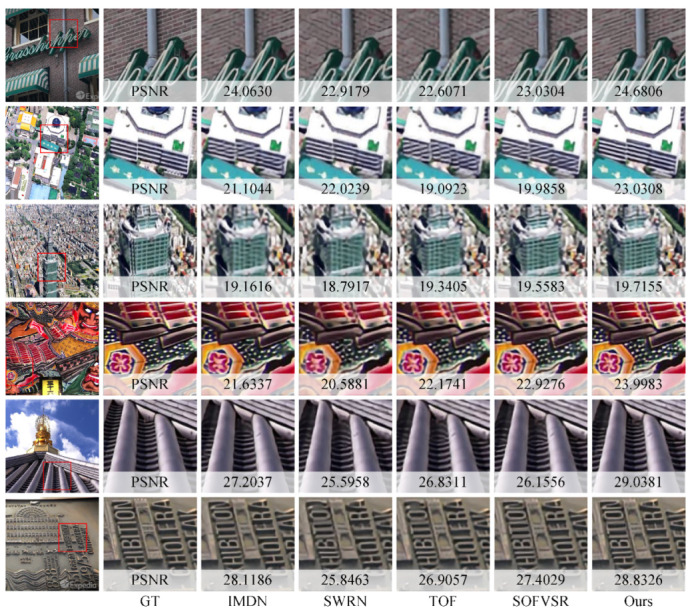
Qualitative Comparisons on the SPMCs-30 Benchmark.

The qualitative comparison is shown in Figure 8, where frames from six videos have been selected for analysis. Arranged from the top row to bottom, the videos are named as follows: AMVTG_004, hdclub_001, hdclub_003, hitachi_isee5, jvc_004, and LDVTG_009. In the case of AMVTG_004, it is evident that all compared models struggle to accurately reproduce the texture of the wall. The GT column is the high-resolution reference. Moreover, some methods result in the presence of undesired artifacts. Similarly, in hdclub_001, only the proposed method and SWRN demonstrate success in recovering the correct structure by effectively leveraging temporal information from neighboring frames. Regrettably, all compared methods exhibit poor performance in hdclub_003. However, the proposed method works well in reconstructing a clear and well-defined structure for both the building and flower in hdclub_003 and hitachi_isee5. The results obtained from jcv_004 show the ability of the proposed method to recover more details. Lastly, the SR frames of LDVTG_009 illustrate how the proposed method effectively utilized the ability of the image SR model, leading to improved results. These qualitative comparisons serve as compelling evidence of the superior performance and effectiveness of the proposed method.

**Figure 9:**
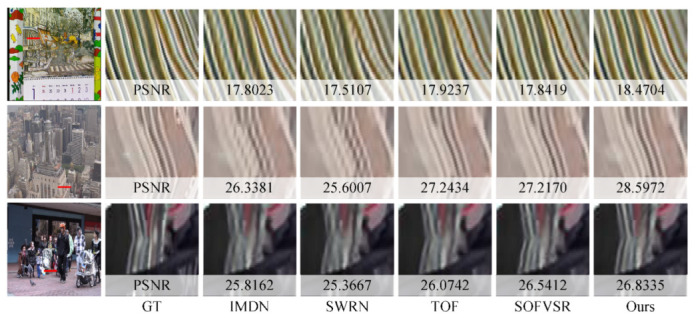
Qualitative Comparisons of Temporal Profiles.

The temporal consistency of the proposed model is evaluated following the methodology in a prior study [33]. The temporal profiles of different methods are shown in Figure 9, with each temporal profile generated at the specified location marked in red, as illustrated in the first column. The reference temporal profile of high-resolution video frames is shown in the GT column. As one can see, the proposed model exhibits superior performance in terms of generating smooth and clearly defined temporal profiles, particularly in Calendar and City. While artifacts are present in the temporal profile of Walk for all methods, the proposed approach demonstrates the fewest instances of such artifacts, indicating its ability to effectively preserve temporal consistency. These findings serve as robust evidence of the enhanced temporal performance of our method.

### 4.4. Efficiency

**Figure 10:**
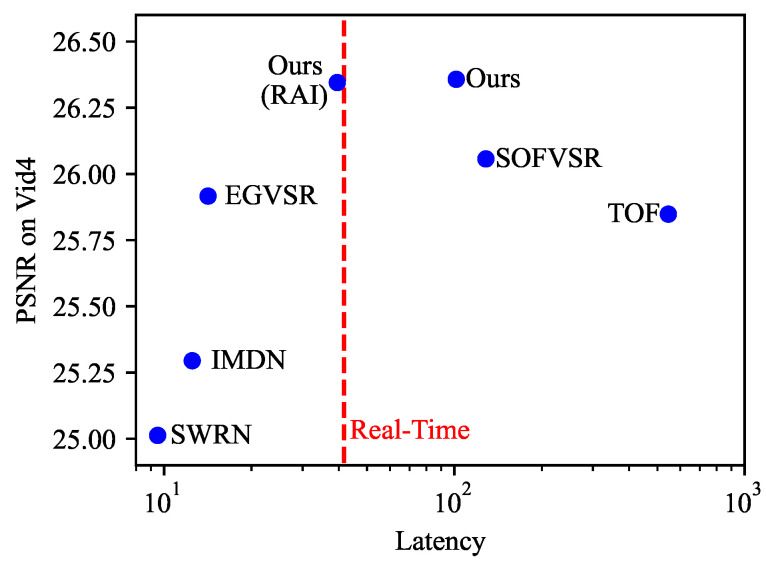
Latency and PSNR on the Vid4 Benchmark.

The efficiency is analyzed from four aspects: number of parameters, number of computational operations, inference latency, and quality of SR results. The float point operations (FLOPs) and latency of each model are evaluated by producing 100 SR frames with a resolution of 1280×720. Further, all models are inferred with a Nvidia 2080ti GPU. The efficiency of the proposed method and compared models are presented in Table 4 and Figure 10. As shown in Table 4, there are four models that are capable of real-time inference. The number of parameters of IMDN [25] and SWRN [19] are relatively small. Further, the small computational complexity of IMDN [25] and SWRN [19] enables real-time inference. However, their PSNR performance is slightly lower than other methods. TOF [7] and SOFVSR [30] need more time for optical flow estimation, so they cannot achieve real-time inference. EGVSR [20] has more parameters than the proposed method. The proposed method performs well in terms of parameter count and PSNR, but it cannot achieve real-time inference due to redundancy. With the integration of the RAI, both latency and FLOPs drop significantly, leading the proposed method to produce real-time 720P SR frames while still achieving competitive performance. These results indicate that the RAI demonstrates an efficient and simple yet effective strategy to optimize the inference process by avoiding unnecessary computations. It achieves a balance between effectiveness and efficiency. Further, the modular design allows it to be integrated into other video models that require spatio-temporal feature extraction.

### 4.5. Ablation Analysis

In this section, ablation studies are presented to examine the impact of key components. IMDN establishes a baseline for comparison, which takes one LR frame as input. Subsequently, the spatial aggregation and temporal aggregation are evaluated. They are key stages in the fast temporal information aggregation module. For measuring the performance of the model with spatial aggregation only, the spatially aggregated features are fused using concatenation and a 3×3 convolutional layer. Table 5 provides the ablation studies of the proposed model, with the second and third columns specifically highlighting the variation.

On the Vid4 benchmark, the baseline model without temporal information achieves a PSNR result of 25.3254 dB and an SSIM result of 72.49%. By incorporating spatial aggregation, there is a noticeable improvement of 0.6499 dB and 3.96% in terms of PSNR and SSIM. Notably, the temporal aggregation in this variation is a simple 3×3 convolution. When the proposed temporal aggregation approach is employed, there is a further increase in performance, with an additional enhancement of 0.315 dB and 1.63% in terms of PSNR and SSIM, respectively. These results validate the significant contributions of both spatial and temporal aggregation components within our method.

Furthermore, an additional analysis is conducted to evaluate the impact of well-trained parameters from the image SR model on the video SR task. As shown in Table 5, the fourth column indicates whether the model was initialized with well-trained image SR parameters. The results demonstrate the significance of utilizing well-trained parameters in the video SR task. Model 4 exhibits superior performance compared to Model 1, while Model 5 outperforms Model 3. These findings suggest that incorporating well-trained parameters from an image SR model can effectively enhance the overall performance of the video SR task. This analysis further emphasizes the importance of leveraging existing knowledge and expertise in the field of image SR to improve the efficiency and effectiveness of video SR models.

### 4.6. Limitation

Although the proposed method can infer 720P video frames in real-time, there are some limitations. First, the LR video frame is synthesized by bicubic degradation. This may deviate from the degradation of actual low-resolution video. Secondly, the performance of the proposed method can be further improved. In Section 4.3, the proposed method achieves the overall best performance. However, it performs worse than IMDN in some videos. In addition, some reconstruction results are not very sharp, for example, “Sunday” and “Monday” in Figure 7. Thirdly, the time consumption is close to the boundary of real-time inference. There is still more room for improvement.

## 5. Conclusions

In this paper, a novel approach for real-time video super-resolution is presented. The method incorporates a pre-trained image super-resolution model as its foundational framework to effectively exploit spatial information. To further leverage inter-frame dependencies, a fast temporal information aggregation module is introduced with the utilization of deformable convolution. This temporal modeling extracts motion cues across frames to enrich the spatial details. Additionally, a redundancy-aware inference algorithm is developed to minimize redundant computations by reusing intermediate features. It reduces the inference latency, enabling real-time performance for 720p video super-resolution with a minimal impact on accuracy. Experiments on several benchmarks show that the proposed method produces high-quality SR results quantitatively and qualitatively. The real-time inference capability makes the proposed method suitable for practical applications requiring live video enhancement. In the future, the efficient video super-resolution approaches can be improved by but not limited to the following directions: advanced degradation model for real-world low-resolution video; attention mechanism for better spatial-temporal feature extraction; novel techniques for efficient inference.

## Figures and Tables

**Table 1:** Details of the Temporal Aggregation Stage.

Layer No.	Input Layer No.	Layer	Input Channels	Output Channels
0		Input		1×
1	0	Concatenation	(2n+1)×	(2n+1)×
2	1	Channel Attention	(2n+1)×	(2n+1)×
3	1 and 2	Elementwise Add	(2n+1)×	(2n+1)×
4	3	Convolution 3×3	(2n+1)×	2×
5	4	RCAB	2×	2×
6	5	LeakyReLU	2×	2×
7	6	Channel Attention	2×	2×
8	6 and 7	Elementwise Add	2×	2×
9	8	Convolution 3×3	2×	1×
10	9	RCAB	1×	1×
11	10	LeakyReLU	1×	1×
12	11	Output	1×	

**Table 2:** Quantitative Comparison on the Vid4 Benchmark. The best and second-best results are marked in red and blue, respectively.

Method	Calendar	City	Foliage	Walk	Average
IMDN [25]	22.1185	25.9733	24.6737	28.4131	25.2947
	0.7078	0.6811	0.6564	0.8693	0.7287
SWRN [19]	21.7028	25.8976	24.4783	27.9725	25.0128
	0.6749	0.6731	0.6458	0.8585	0.7131
3DSRnet [31]	22.5174	27.1086	25.5571	27.7540	25.7433
	0.6586	0.6987	0.6898	0.8681	0.7288
TOF [7]	22.4371	26.6647	25.3451	28.9459	25.8482
	0.7242	0.7356	0.707	0.8799	0.7617
EGVSR [20]	23.5585	27.4242	24.7348	27.9476	25.9163
	0.7959	0.8009	0.7091	0.8599	0.7915
SOFVSR [30]	22.7644	26.8175	25.5315	29.1136	26.0568
	0.7463	0.7495	0.7182	0.8835	0.7744
RISTN [42]	22.9171	26.9975	25.5761	29.2238	26.1786
	0.7504	0.7582	0.7221	0.8814	0.7780
Ours	23.0747	27.0412	25.6840	29.6298	26.3574
	0.7626	0.7642	0.7255	0.8937	0.7865
Ours (RAI)	23.0682	27.0244	25.6730	29.6147	26.3451
	0.7621	0.7627	0.7248	0.8935	0.7858

**Table 3:** Quantitative Comparison on the SPMCs-30 Benchmark. The best and second-best results are marked in red and blue, respectively.

Method	IMDN [25]	SWRN [19]	TOF [7]	EGVSR [20]	SOFVSR [30]	RISTN [42]	Ours	Ours (RAI)
AMVTG_004	26.0314	24.9373	24.8697	22.7946	25.1903	25.6177	26.4554	26.4566
	0.7179	0.6398	0.6442	0.5583	0.6643	0.6820	0.7456	0.7456
HKVTG_004	28.5451	28.2554	28.4698	25.3357	28.6597	28.6793	28.8824	28.8816
	0.7519	0.7391	0.7483	0.6234	0.7596	0.7597	0.7694	0.7693
LDVTG_009	26.8267	25.7857	26.4415	27.1797	27.0993	27.6783	27.7354	27.7327
	0.8353	0.8052	0.8345	0.8551	0.8501	0.8588	0.8653	0.8653
LDVTG_022	29.7429	29.2831	29.3073	27.1004	29.4682	29.8795	30.1291	30.1330
	0.8496	0.8352	0.8392	0.7822	0.8432	0.8502	0.8604	0.8605
NYVTG_006	29.4499	29.2991	30.2000	25.9653	30.9459	30.6516	30.9080	30.9066
	0.8564	0.8452	0.8599	0.7647	0.8785	0.8723	0.8819	0.8818
PRVTG_008	23.8790	23.4167	23.8181	21.1342	24.1036	24.2942	24.5739	24.5743
	0.6764	0.6534	0.6741	0.5533	0.6959	0.7067	0.7167	0.7166
PRVTG_012	26.4371	26.2694	26.5504	24.5646	26.7048	26.8525	26.9413	26.9435
	0.7718	0.7628	0.7750	0.7129	0.7839	0.7899	0.7945	0.7945
RMVTG_011	25.8581	25.4017	25.9722	23.8420	26.2772	26.5048	26.6950	26.6929
	0.7458	0.7254	0.7488	0.6703	0.7642	0.7711	0.7797	0.7795
RMVTG_024	25.2832	24.9563	25.3642	23.5458	25.7016	25.8718	25.9665	25.9703
	0.6664	0.6488	0.6720	0.6162	0.6972	0.7103	0.7109	0.7109
TPVTG_003	30.3131	29.7610	29.9361	27.3018	29.9714	30.3205	30.7238	30.7237
	0.8815	0.8687	0.8725	0.7860	0.8753	0.8773	0.8898	0.8898
cact1_001	32.2501	31.1136	32.1522	31.3228	32.3432	32.5230	33.3811	33.3668
	0.9075	0.8904	0.9196	0.9111	0.9214	0.9178	0.9284	0.9281
car05_001	29.5382	29.1795	30.0287	28.9618	30.1839	29.8935	30.0922	30.0779
	0.8423	0.8338	0.8620	0.8309	0.8637	0.8423	0.8537	0.8532
gree3_001	29.9863	29.6108	29.6615	26.9135	29.9342	30.1444	30.3567	30.3561
	0.8119	0.8030	0.8102	0.7017	0.8180	0.8163	0.8249	0.8248
hdclub_001	23.8864	23.4446	23.8494	22.8246	24.0650	24.5185	24.8958	24.9024
	0.7387	0.7148	0.7484	0.7338	0.7603	0.7756	0.7869	0.7870
hdclub_003	20.3518	20.1453	20.8639	19.3091	21.0255	21.2868	21.1774	21.1785
	0.6015	0.5881	0.6535	0.6207	0.6679	0.6896	0.6751	0.6752
hdclub_008	25.9392	25.7197	26.1188	24.6306	26.2198	26.2789	26.4213	26.4210
	0.7184	0.7045	0.7312	0.6647	0.7369	0.7381	0.7473	0.7473
hitachi_isee5	23.1258	21.9516	22.9783	24.6103	23.3418	24.1477	24.3415	24.3261
	0.8079	0.7568	0.8055	0.8655	0.8187	0.8358	0.8470	0.8464
hk001_001	29.6545	29.1030	29.5680	26.7095	29.8456	30.1093	30.4816	30.4851
	0.7580	0.7459	0.7758	0.6702	0.7845	0.7868	0.7949	0.7948
hk004_006	30.8958	30.1270	30.5283	28.1557	31.0959	31.1093	31.7502	31.7509
	0.8463	0.8324	0.8559	0.8003	0.8634	0.8616	0.8719	0.8719
indi1_004	33.0746	32.2322	32.8443	31.7546	33.0945	33.4130	34.2882	34.2895
	0.8891	0.8709	0.8913	0.8870	0.8976	0.8988	0.9145	0.9145
indi1_032	34.6416	33.4208	34.4735	33.4178	34.5877	34.8725	36.2915	36.2836
	0.9246	0.9077	0.9315	0.9201	0.9352	0.9303	0.9469	0.9467
jvc_004	29.8523	28.7227	30.0097	31.6536	29.9930	30.6357	31.0591	31.0441
	0.9494	0.9329	0.9513	0.9632	0.9502	0.9531	0.9612	0.9611
jvc_009	27.7313	27.1172	27.7947	26.8302	28.0539	28.0865	28.6781	28.6749
	0.8500	0.8263	0.8517	0.8462	0.8606	0.8571	0.8758	0.8756
land5_001	37.0995	35.7070	36.0325	34.8101	35.9214	36.7371	38.2512	38.2421
	0.9602	0.9548	0.9637	0.9573	0.9647	0.9578	0.9681	0.9680
land9_007	34.9431	33.9314	34.4512	32.2815	34.7169	35.0958	36.1717	36.1766
	0.9161	0.9094	0.9288	0.8839	0.9297	0.9264	0.9360	0.9361
philips_hkc01	35.6114	33.9995	34.2302	34.6706	34.9984	34.7056	36.2910	36.2628
	0.9373	0.9104	0.9125	0.9254	0.9274	0.9193	0.9429	0.9426
philips_hkc04	34.2978	33.5183	34.1432	31.0672	32.6543	32.8824	34.2802	34.2844
	0.8927	0.8797	0.8917	0.8267	0.8712	0.8624	0.8879	0.8880
philips_hkc05	30.3882	29.3879	30.8576	32.1182	30.8332	30.7311	31.2771	31.2218
	0.8448	0.8052	0.8576	0.9017	0.8587	0.8541	0.8711	0.8692
philips_hkc11	36.4101	35.2255	35.7448	32.6174	35.6057	35.5644	36.8548	36.8287
	0.8961	0.8713	0.8846	0.8316	0.8850	0.8810	0.9042	0.9036
veni3_011	33.1165	32.2105	32.7536	29.8624	33.1524	33.1738	34.5723	34.5763
	0.9562	0.9444	0.9527	0.9047	0.9571	0.9524	0.9645	0.9646
Average	29.5054	28.7745	29.3338	27.7762	29.5263	29.7505	30.3308	30.3255
	0.8267	0.8069	0.8283	0.7856	0.8361	0.8378	0.8506	0.8504

**Table 4:** Quantitative Comparison of Efficiency for Producing 720P Frames.

Method	Parameters	FLOPs	Latency (ms)	Real-Time Inference	PSNR on Vid4	PSNR on SPMCs-30
IMDN [25]	715K	40.91G	12.50	✓	25.2947	29.5054
SWRN [19]	43K	5.00G	9.50	✓	25.0128	28.7744
TOF [7]	1405K	133.06G	545.77	✕	25.8482	29.3338
EGVSR [20]	2587K	102.89G	14.17	✓	25.9163	27.7762
SOFVSR [30]	1048K	120.83G	128.36	✕	26.0568	29.5263
Ours	1895K	336.40G	101.47	✕	26.3574	30.3308
Ours (RAI)	1895K	109.07G	39.51	✓	26.3451	30.3255

**Table 5:** Quantitative Performance for the Ablation Study.

Model	Spatial Aggregation	Temporal Aggregation	Pre-Trained Parameters	Vid4		SPMCs-30	
				**PSNR**	**SSIM**	**PSNR**	**SSIM**
Model 1	No	No	No	25.3254	0.7249	29.3972	0.8216
Model 2	Yes	No	No	25.9753	0.7645	29.6212	0.8317
Model 3	Yes	Yes	No	26.2903	0.7808	30.0463	0.8429
Model 4	No	No	Yes	25.4421	0.7318	29.6574	0.8288
Model 5	Yes	Yes	Yes	26.3574	0.7865	30.3308	0.8506

## Data Availability

The public data used in this work are listed here: Vimeo90k (toflow.csail.mit.edu) http://toflow.csail.mit.edu/index.html#septuplet (accessed on 12 December 2022), Vid4 (Google Drive) https://drive.google.com/file/d/1ZuvNNLgR85TV_whJoHM7uVb-XW1y70DW/view?usp=sharing (accessed on 12 December 2022), and SPMCs-30 (GitHub) https://github.com/jiangsutx/SPMC_VideoSR (accessed on 12 December 2022).

## References

[1] Kappeler A., Yoo S., Dai Q., Katsaggelos A.K. (2016). Video Super-Resolution With Convolutional Neural Networks. IEEE Trans. Comput. Imaging.

[2] Rota C., Buzzelli M., Bianco S., Schettini R. (2023). Video restoration based on deep learning: A comprehensive survey. Artif. Intell. Rev..

[3] Farooq M., Dailey M.N., Mahmood A., Moonrinta J., Ekpanyapong M. (2021). Human face super-resolution on poor quality surveillance video footage. Neural Comput. Appl..

[4] Xiao Y., Su X., Yuan Q., Liu D., Shen H., Zhang L. (2022). Satellite Video Super-Resolution via Multiscale Deformable Convolution Alignment and Temporal Grouping Projection. IEEE Trans. Geosci. Remote Sens..

[5] Anwar S., Khan S.H., Barnes N. (2020). A Deep Journey into Super-resolution: A Survey. ACM Comput. Surv..

[6] Jo Y., Oh S.W., Kang J., Kim S.J. (2018). Deep Video Super-Resolution Network Using Dynamic Upsampling Filters without Explicit Motion Compensation. Proceedings of the 2018 IEEE Conference on Computer Vision and Pattern Recognition, CVPR 2018, Salt Lake City, UT, USA, 18–22 June 2018.

[7] Xue T., Chen B., Wu J., Wei D., Freeman W.T. (2019). Video Enhancement with Task-Oriented Flow. Int. J. Comput. Vis..

[8] Wang X., Chan K.C.K., Yu K., Dong C., Loy C.C. (2019). EDVR: Video Restoration With Enhanced Deformable Convolutional Networks. Proceedings of the IEEE Conference on Computer Vision and Pattern Recognition Workshops, CVPR Workshops 2019, Long Beach, CA, USA, 16–20 June 2019.

[9] Choi Y.J., Lee Y., Kim B. (2020). Wavelet Attention Embedding Networks for Video Super-Resolution. Proceedings of the 25th International Conference on Pattern Recognition, ICPR 2020, Milan, Italy, 10–15 January 2021.

[10] Liang J., Fan Y., Xiang X., Ranjan R., Ilg E., Green S., Cao J., Zhang K., Timofte R., Gool L.V. Recurrent Video Restoration Transformer with Guided Deformable Attention. Proceedings of the Advances in Neural Information Processing Systems 35: Annual Conference on Neural Information Processing Systems 2022.

[11] Caballero J., Ledig C., Aitken A.P., Acosta A., Totz J., Wang Z., Shi W. (2017). Real-Time Video Super-Resolution with Spatio-Temporal Networks and Motion Compensation. Proceedings of the 2017 IEEE Conference on Computer Vision and Pattern Recognition, CVPR 2017, Honolulu, HI, USA, 21–26 July 2017.

[12] Chan K.C.K., Wang X., Yu K., Dong C., Loy C.C. (2021). BasicVSR: The Search for Essential Components in Video Super-Resolution and Beyond. Proceedings of the IEEE Conference on Computer Vision and Pattern Recognition, CVPR 2021, Virtual, 19–25 June 2021.

[13] Bao W., Lai W., Zhang X., Gao Z., Yang M. (2021). MEMC-Net: Motion Estimation and Motion Compensation Driven Neural Network for Video Interpolation and Enhancement. IEEE Trans. Pattern Anal. Mach. Intell..

[14] Tao X., Gao H., Liao R., Wang J., Jia J. (2017). Detail-Revealing Deep Video Super-Resolution. Proceedings of the IEEE International Conference on Computer Vision, ICCV 2017, Venice, Italy, 22–29 October 2017.

[15] Yi P., Wang Z., Jiang K., Jiang J., Ma J. (2019). Progressive Fusion Video Super-Resolution Network via Exploiting Non-Local Spatio-Temporal Correlations. Proceedings of the 2019 IEEE/CVF International Conference on Computer Vision, ICCV 2019, Seoul, Republic of Korea, 27 October–2 November 2019.

[16] Li W., Tao X., Guo T., Qi L., Lu J., Jia J., Vedaldi A., Bischof H., Brox T., Frahm J. (2020). MuCAN: Multi-correspondence Aggregation Network for Video Super-Resolution. Proceedings of the Computer Vision—ECCV 2020—16th European Conference, Glasgow, UK, 23–28 August 2020.

[17] Li S., He F., Du B., Zhang L., Xu Y., Tao D. (2019). Fast Spatio-Temporal Residual Network for Video Super-Resolution. Proceedings of the IEEE Conference on Computer Vision and Pattern Recognition, CVPR 2019, Long Beach, CA, USA, 16–20 June 2019.

[18] Xia B., He J., Zhang Y., Wang Y., Tian Y., Yang W., Van Gool L. Structured Sparsity Learning for Efficient Video Super-Resolution. Proceedings of the 2023 IEEE/CVF Conference on Computer Vision and Pattern Recognition (CVPR).

[19] Lian W., Lian W., Karlinsky L., Michaeli T., Nishino K. (2022). Sliding Window Recurrent Network for Efficient Video Super-Resolution. Proceedings of the Computer Vision—ECCV 2022 Workshops—Tel Aviv, Israel, 23–27 October 2022.

[20] Cao Y., Wang C., Song C., Tang Y., Li H. (2021). Real-Time Super-Resolution System of 4K-Video Based on Deep Learning. Proceedings of the 32nd IEEE International Conference on Application-Specific Systems, Architectures and Processors, ASAP 2021, Virtual Conference, 7–9 July 2021.

[21] Dai J., Qi H., Xiong Y., Li Y., Zhang G., Hu H., Wei Y. (2017). Deformable Convolutional Networks. Proceedings of the IEEE International Conference on Computer Vision, ICCV 2017, Venice, Italy, 22–29 October 2017.

[22] Zhang Y., Li K., Li K., Wang L., Zhong B., Fu Y., Ferrari V., Hebert M., Sminchisescu C., Weiss Y. (2018). Image Super-Resolution Using Very Deep Residual Channel Attention Networks. Proceedings of the Computer Vision—ECCV 2018—15th European Conference, Munich, Germany, 8–14 September 2018.

[23] Dong C., Loy C.C., He K., Tang X. (2016). Image Super-Resolution Using Deep Convolutional Networks. IEEE Trans. Pattern Anal. Mach. Intell..

[24] Lim B., Son S., Kim H., Nah S., Lee K.M. (2017). Enhanced Deep Residual Networks for Single Image Super-Resolution. Proceedings of the 2017 IEEE Conference on Computer Vision and Pattern Recognition Workshops, CVPR Workshops 2017, Honolulu, HI, USA, 21–26 July 2017.

[25] Hui Z., Gao X., Yang Y., Wang X., Amsaleg L., Huet B., Larson M.A., Gravier G., Hung H., Ngo C., Ooi W.T. (2019). Lightweight Image Super-Resolution with Information Multi-distillation Network. Proceedings of the Proceedings of the 27th ACM International Conference on Multimedia, MM 2019, Nice, France, 21–25 October 2019.

[26] Vaswani A., Shazeer N., Parmar N., Uszkoreit J., Jones L., Gomez A.N., Kaiser L., Polosukhin I. Attention is All you Need. Proceedings of the Advances in Neural Information Processing Systems 30: Annual Conference on Neural Information Processing Systems 2017.

[27] Dosovitskiy A., Beyer L., Kolesnikov A., Weissenborn D., Zhai X., Unterthiner T., Dehghani M., Minderer M., Heigold G., Gelly S. An Image is Worth 16x16 Words: Transformers for Image Recognition at Scale. Proceedings of the 9th International Conference on Learning Representations, ICLR 2021.

[28] Liu Z., Lin Y., Cao Y., Hu H., Wei Y., Zhang Z., Lin S., Guo B. (2021). Swin Transformer: Hierarchical Vision Transformer using Shifted Windows. Proceedings of the 2021 IEEE/CVF International Conference on Computer Vision, ICCV 2021, Montreal, QC, Canada, 10–17 October 2021.

[29] Liang J., Cao J., Sun G., Zhang K., Gool L.V., Timofte R. (2021). SwinIR: Image Restoration Using Swin Transformer. Proceedings of the IEEE/CVF International Conference on Computer Vision Workshops, ICCVW 2021, Montreal, BC, Canada, 11–17 October 2021.

[30] Wang L., Guo Y., Liu L., Lin Z., Deng X., An W. (2020). Deep Video Super-Resolution Using HR Optical Flow Estimation. IEEE Trans. Image Process..

[31] Kim S.Y., Lim J., Na T., Kim M. (2019). Video Super-Resolution Based on 3D-CNNS with Consideration of Scene Change. Proceedings of the 2019 IEEE International Conference on Image Processing, ICIP 2019, Taipei, Taiwan, 22–25 September 2019.

[32] Isobe T., Li S., Jia X., Yuan S., Slabaugh G.G., Xu C., Li Y., Wang S., Tian Q. (2020). Video Super-Resolution With Temporal Group Attention. Proceedings of the 2020 IEEE/CVF Conference on Computer Vision and Pattern Recognition, CVPR 2020, Seattle, WA, USA, 13–19 June 2020.

[33] Tian Y., Zhang Y., Fu Y., Xu C. (2020). TDAN: Temporally-Deformable Alignment Network for Video Super-Resolution. Proceedings of the 2020 IEEE/CVF Conference on Computer Vision and Pattern Recognition, CVPR 2020, Seattle, WA, USA, 13–19 June 2020.

[34] Ying X., Wang L., Wang Y., Sheng W., An W., Guo Y. (2020). Deformable 3D Convolution for Video Super-Resolution. IEEE Signal Process. Lett..

[35] Xiao Y., Yuan Q., Jiang K., Jin X., He J., Zhang L., Lin C. (2023). Local-Global Temporal Difference Learning for Satellite Video Super-Resolution. arXiv.

[36] Wang H., Xiang X., Tian Y., Yang W., Liao Q. (2023). STDAN: Deformable Attention Network for Space-Time Video Super-Resolution. IEEE Trans. Neural Netw. Learn. Syst..

[37] Xiao Y., Yuan Q., Zhang Q., Zhang L. (2023). Deep Blind Super-Resolution for Satellite Video. IEEE Trans. Geosci. Remote Sens..

[38] Shi S., Gu J., Xie L., Wang X., Yang Y., Dong C., Koyejo S., Mohamed S., Agarwal A., Belgrave D., Cho K., Oh A. (2022). Rethinking Alignment in Video Super-Resolution Transformers. Proceedings of the Advances in Neural Information Processing Systems 35: Annual Conference on Neural Information Processing Systems 2022, New Orleans, LA, USA, 28 November–9 December 2022.

[39] Kingma D.P., Ba J. Adam: A Method for Stochastic Optimization. Proceedings of the 3rd International Conference on Learning Representations—ICLR 2015.

[40] Agustsson E., Timofte R. NTIRE 2017 Challenge on Single Image Super-Resolution: Dataset and Study. Proceedings of the The IEEE Conference on Computer Vision and Pattern Recognition (CVPR) Workshops.

[41] Wang Z., Bovik A., Sheikh H., Simoncelli E. (2004). Image quality assessment: From error visibility to structural similarity. IEEE Trans. Image Process..

[42] Zhu X., Li Z., Zhang X.Y., Li C., Liu Y., Xue Z. (2019). Residual Invertible Spatio-Temporal Network for Video Super-Resolution. Proc. AAAI Conf. Artif. Intell..

